# The Kirkwood–Riseman Model of Polymer Solution Dynamics Is Qualitatively Correct

**DOI:** 10.3390/polym15091995

**Published:** 2023-04-23

**Authors:** George David Joseph Phillies

**Affiliations:** Department of Physics, Worcester Polytechnic Institute, Worcester, MA 01609-2280, USA; phillies@4liberty.net; Tel.: +1-508-754-1859

**Keywords:** polymer dynamics, molecular dynamics, Brownian dynamics, Rouse model, Kirkwood–Riseman model, computer simulation, Rouse modes

## Abstract

The Rouse model is the foundational basis of much of modern polymer physics. The period alternative, the Kirkwood–Riseman model, is rarely mentioned in modern monographs. The models are qualitatively different. The models do not agree as to how many internal modes a polymer molecule has. In the Kirkwood–Riseman model, polymers in a shear field perform whole-body rotation; in the Rouse model, polymers respond to shear with an affine deformation. We use Brownian dynamics to show that the Kirkwood–Riseman model for chain motion is qualitatively correct. Contrary to the Rouse model, in shear flow, polymer coils rotate. Rouse modes are cross-correlated. The amplitudes and relaxation rates of Rouse modes depend on the shear rate. Several alternatives to Rouse modes as collective coordinates are discussed.

## 1. Introduction

Seven decades ago, Kirkwood and Riseman [1], Rouse [2], and Zimm [3] advanced simple, seemingly transparent models for the dynamics of dilute polymers in solution. The models were similar in that each approximated a polymer coil as a series of hydrodynamically active points (“beads”) held together by hydrodynamically inert connectors (“springs”). A particular focus of their models was a calculation of the polymeric contribution to a solution’s viscosity. Here, the similarity between the models ends. As is not uniformly recognized, the Kirkwood–Riseman and Rouse–Zimm models give contradictory descriptions of how polymer chains move in solution and contribute to the solution’s viscosity. According to Rouse, a polymer coil responds to a shear flow with a non-rotational affine deformation of the polymer’s shape. According to Kirkwood and Riseman, a polymer coil responds to a shear flow by performing whole-body rotation.

In the Rouse and Zimm models, the connectors (“springs”) between the beads create Hooke’s-law restoring forces that pull the beads together. Thermal fluctuations in the solvent create random forces on the polymer beads, the random forces tending on the average to drive the beads apart. The competition between the spring and thermal forces determines the size of a polymer coil. In the absence of thermal forces, a Rouse chain contracts to a point. Rouse–Zimm polymer coils can translate; all other bead motions are described by internal (Rouse) modes, in which the relative positions of the beads change. These internal modes are said by Rouse to be responsible for the polymer coil’s contribution to the viscosity and for the relaxation of the polymer’s end-to-end vector, which in some polymers can be tracked with dielectric relaxation.

The Kirkwood–Riseman model invokes three fundamental assumptions. First, all distances between pairs of beads are treated as being their statistico-mechanical average values; fluctuations and changes in these distances are explicitly not included in the model. Second, the distribution of beads around the chain center-of-mass is *on the average* spherically symmetric. Third, the system is massively overdamped, so that the inertia of the polymer coil is negligible. These three assumptions completely define the chain dynamics, the description of how a Kirkwood–Riseman polymer chain moves in solution. In the Kirkwood–Riseman model, the dominant contributions to a polymer’s intrinsic viscosity and diffusion coefficient arise from whole-body translation and rotation. While Kirkwood and Riseman recognized that polymer beads in a single chain do move with respect to each other, so that polymer chains do have segmental dynamics, within their model these internal motions are neglected.

Polymer models make different assumptions as to the role of hydrodynamic interactions between different parts of the polymer chain. In free-draining models, such as the Rouse model, bead–bead hydrodynamic interactions are absent. In non-draining models, such as the Zimm model, polymer beads have hydrodynamic interactions, described in the Zimm model by the Oseen hydrodynamic interaction tensor. The Kirkwood–Riseman model has two parts: In the calculation of the intrinsic viscosity, the chain is taken to be non-draining. In the calculation of how the chain moves in response to an applied shear field, the model may be equally said to be free-draining or non-draining, because Newton’s Third Law of Motion guarantees that internal (here, hydrodynamic) forces have no effect on polymeric motions within the constraints of the model.

This paper presents a simulational test of the Rouse and Kirkwood–Riseman descriptions of polymer motion. We examine an isolated chain in a hydrodynamic shear field. The chain moves in accord with Rouse’s dynamic equations of motion, as described below and in the Supplementary Materials, with the addition of a shear field and random thermal forces on each bead. We employ a wider range of computational diagnostics than has sometimes been used in the past to interpret polymer dynamics. In particular, we ask whether or not a chain in a shear field rotates. According to the Rouse model, fluctuations in the amplitudes of different Rouse modes are not cross-correlated, while relaxation rates and mean-square amplitudes of Rouse modes are independent of the shear rate. We therefore ask if fluctuations in Rouse mode amplitudes are cross-correlated, and if Rouse mode amplitudes or relaxation rates depend on the applied shear rate. We demonstrate two sets of alternative diagnostics, namely spatial Fourier transforms and Haar wavelets [4], for characterizing polymer motion.

The next Section of the paper describes our simulation procedures, including the physical quantities that we calculated. We then outline our major results, revealing the relative validities of the Rouse and Kirkwood–Riseman models as descriptions of polymer dynamics. To anticipate our results, we show that Kirkwood and Riseman were correct, and Rouse was incorrect; polymer chains in shear rotate; they do not displace via an affine deformation. Details of the Rouse and Kirkwood–Riseman models are found in this paper’s Supplementary Materials.

## 2. Material and Methods

A Brownian dynamics simulation for a polymer in a shear field was implemented. The equations of motion resemble the Rouse equations of motion, but a shear field has been added. The simulations reveal how a Rouse-like polymer coil moves in a rheological experiment, as envisioned by Rouse and also by Kirkwood and Riseman. As the Rouse model is largely familiar, a detailed description is supplied in the Supplementary Materials.

For beads i∈(2,…,N−1), we write the equations of motion as

(1) $$\frac{dR_{i}}{dt}=f^{-1}\left(-k\left(2R_{i}-R_{i-1}-R_{i+1}\right)\right)+Gy_{i}\hat{i}+F_{i}\left(t\right),$$

$\hat{i}$ being the unit vector in the *x*-direction, *G* being the shear in the supporting medium, and $F_{i}\left(t\right)$ being the time-dependent random thermal force. For beads 1 and *N*, the equations of motion are

(2) $$\frac{dR_{1}}{dt}=f^{-1}\left(-k\left(R_{1}-R_{2}\right)\right)+Gy_{1}\hat{i}+F_{1}\left(t\right),$$

and

(3) $$\frac{dR_{N}}{dt}=f^{-1}\left(-k\left(N_{i}-R_{N-1}\right)\right)+Gy_{N}\hat{i}+F_{N}\left(t\right).$$

These equations describe chain motion relative to the chain center. The shear force $Gy_{i}\hat{i}$ is directed in the $\hat{i}$ direction, and changes linearly with the distance in the *y*-direction from the chain center-of-mass.

The above are a set of *N*-coupled linear differential equations whose coefficients are constants. For G=0 and $F_{N}\left(t\right)=0$, the solutions are the 3N Rouse modes $Q_{n}$, with each mode describing bead motions parallel to one of the coordinate axes. Each mode has a corresponding eigenvalue $\Gamma_{n}$. The bead coordinates $x_{i}\left(t\right)$ determine the normal mode amplitudes $C_{nx}\left(t\right)$ for the *x*-coordinate modes via

(4) $$C_{nx}\left(t\right)=\frac{1}{N}\sum_{i=1}^{N}x_{i}\left(t\right)\cos\left(\frac{n\pi (i-1/2)}{N}\right).$$

Entirely similar equations give the amplitudes $C_{ny}$ and $C_{nz}$ of the *y*- and *z*-coordinate modes. The inverse equations give the $x_{i}$ in terms of the normal mode amplitudes as

(5) $$x_{i}\left(t\right)=C_{0x}\left(t\right)+2\sum_{n=1}^{N-1}C_{nx}\left(t\right)\cos\left(\frac{n\pi (i-1/2)}{N}\right).$$

Entirely similar equations give the *y* and *z* coordinates of the beads.

For G=0 and $F_{N}\left(t\right)=0$, one Rouse mode has eigenvalue $\Gamma_{0}=0$; that mode corresponds to the center-of-mass location of the polymer, its time derivative representing uniform translation of all beads with the same velocity. The other N−1 modes decay exponentially ($\exp(-\Gamma_{n}t)$) in time; their relaxation rates $\Gamma_{n}$ are

(6) $$\Gamma_{n}=\frac{8k\sin^{2}\left(n\pi /2N\right)}{f}$$

with n∈(1,N−1) being the mode label.

The random forces $F_{xi}\left(t\right)$ serve as source terms, driving the fluctuations in the $C_{nx}\left(t\right)$. In the absence of a shear force or a random force, beads move by converging on the chain center of mass. The Rouse model thus has three translational modes, each with eigenvalue zero, and 3N−3 internal modes (`internal’ in the sense that in each internal mode the beads move with respect to each other as time goes on) having non-zero eigenvalues. Rouse applied the Rouse modes, which describe polymer motion in the absence of shear, to calculate viscous dissipation in the presence of shear, the absence or presence of shear being a rarely-mentioned disjuncture in the calculation.

Polymer coils whose motions are described by Rouse’s model have one ill-recognized property: They do not rotate. This property follows by comparison with a standard problem in classical mechanics, namely the vibrational modes of an isolated molecule. In general, an *N*-atom molecule has 3 translational modes with eigenvalue zero, 3 rotational modes with eigenvalue zero, and 3N−6 internal vibrational modes. The internal modes are the modes that change the distances between pairs of atoms. In translation and rotation, the distances between the atoms remain fixed. The Rouse problem only differs from the molecular vibration problem in that the Rouse equations of motion are overdamped, so the Rouse amplitudes relax exponentially at some rate $\Gamma_{n}$ rather than oscillating at some frequency $\omega_{n}$. A polymer coil is therefore like a vibrating isolated molecule in having a total of 3N modes. However, the 3N modes of the Rouse model include 3 translational modes and 3N−3 internal modes, for a total of 3N modes, leaving no modes available for rotational motion.

The statement that Rouse chains do not rotate is not new. Rouse specifies in his paper that a polymer coil under shear does not rotate, namely (his paper, p. 1274, column 2) “…since the velocity of the liquid has a nonvanishing component only in the *x* direction, the components ${\left({\dot{y}}_{j}\right)}_{\alpha }$ and ${\left({\dot{z}}_{j}\right)}_{\alpha }$ are zero”. ${\left({\dot{y}}_{j}\right)}_{\alpha }$ and ${\left({\dot{z}}_{j}\right)}_{\alpha }$ are the velocities of bead *j* in the *y* and *z* directions due to the shear. If the chain is rotating, either ${\left({\dot{y}}_{j}\right)}_{\alpha }$ or ${\left({\dot{z}}_{j}\right)}_{\alpha }$ must be non-zero. Rouse also argues in his paper (p. 1274, column 2, top) that ‘…an atom at the junction between two submolecules…’ (springs) moves ‘…with a velocity equal to that of the surrounding liquid…’ except for Brownian motion, because, according to Rouse, otherwise there would be motion of the solvent relative to the polymer chain, leading to energy dissipation. If the beads only move with the liquid, then they can only be moving parallel to the *x*-axis. According to Rouse, the result of these motions is that a polymer molecular responds to a shear field by making an affine deformation.

In the simulations, the thermal forces $F_{i}\left(t\right)$ were generated using standard methods as independent random variables having Gaussian distributions. The bead displacements during a single time step Δt are $\Delta t\frac{dR_{i}}{dt}$. To compute the trajectory of each bead, the forces are re-evaluated after each time step. Multiple characteristic functions of chain behavior were determined. Most of these functions were used as diagnostics to validate the core software. The radius of gyration and mean-square radius of gyration were calculated. The mean-square center-of-mass displacement was found to be linear in time, as expected. The second $\langle x_{\alpha }x_{\beta }\rangle$ and fourth $\langle x_{\alpha }^{2}x_{\beta }^{2}\rangle$ moments of the bead distribution around the center of mass were calculated. Here, α,β∈(1,3) designate individual Cartesian components of the vectors from the chain center to each bead, the average being over all beads and all times.

Distribution functions for the nearest-neighbor distance, the second-nearest-neighbor distance, the magnitude of the end-to-end vector, the distance from the polymer center-of-mass to each bead, and the distances between all pairs of beads were measured. The time autocorrelation functions $\langle R_{e}\left(0\right)\cdot R_{e}\left(t\right)\rangle$ and $\langle {\hat{R}}_{e}\left(0\right)\cdot {\hat{R}}_{e}\left(t\right)\rangle$ of the chain end-to-end vector $R_{e}=R_{N}-R_{1}$ and its unit vector ${\hat{R}}_{e}=\left(R_{N}-R_{1}\right)/|R_{N}-R_{1})|$ were obtained.

Using Equation (4), we calculated the time-dependent Rouse amplitudes $C_{n\alpha }\left(t\right)$ of the bead positions. For an *N*-bead system, there are 3(N−1) such components, plus the three $C_{0\alpha }$ describing the polymer center-of-mass position. We also calculated several time-dependent spatial Fourier components

(7) $$a_{k,\alpha }\left(t\right)=\sum_{i=1}^{N}\cos\left(kr_{i\alpha }\left(t\right)\right)$$

of the bead locations. Here, *k* is the wavenumber for the transformation and $r_{i\alpha }\left(t\right)$ is the αth Cartesian component of the location of bead *i*, relative to the chain center of mass, at time *t*.

Finally, we calculated the Haar [4] wavelet [5] components c(n,α,j)(t) and d(n,α,j)(t) of the particle positions. In this calculation, *n* is the wavelet decomposition level, α is again the Cartesian coordinate, and *j* labels the wavelet location along the polymer chain. The maximum value of *j* depends on *N*. For a $2^{m}$ bead polymer, the upper limit on *j* is $2^{m-n}$ with m−n≥0. The decomposition proceeds naturally if for some integer *m* there are $2^{m}$ beads in the chain. For n=1, the wavelet components are defined as

(8) $$\begin{array}{ccc} c(1,\alpha ,j) & = & (r_{\alpha ,2*j}+r_{\alpha ,2*j-1})/2 \end{array}$$

(9) $$\begin{array}{ccc} d(1,\alpha ,j) & = & (r_{\alpha ,2*j}-r_{\alpha ,2*j-1})/2; \end{array}$$

for n>1, an iterative definition begins with the above two equations, leading to

(10) $$\begin{array}{ccc} c(n,\alpha ,j) & = & (c(n-1,\alpha ,2*j)+c(n-1,\alpha ,2*j-1)/2 \end{array}$$

(11) $$\begin{array}{ccc} d(n,\alpha ,j) & = & (c(n-1,\alpha ,2*j)-c(n-1,\alpha ,2*j-1))/2. \end{array}$$

With each increment of *n*, the range of allowed values of *j* is cut in half. For each *n*, the ends correspond to the smallest and largest values of *j*. The c(n,α,j) and the d(n,α,j) differ from the spatial Fourier components and the Rouse components in that they are localized; they refer to the behavior of specific parts of the polymer coil. In contrast, the $a_{k,\alpha }\left(t\right)$ and the $C_{n\alpha }\left(t\right)$ are global variables, each depending on the relative positions of all the beads in the chain.

For the $a_{\alpha ,k}\left(t\right)$, the $C_{n\alpha }\left(t\right)$, and the d(n,α,j)(t), the temporal self-correlation functions were evaluated. For the $a_{\alpha ,k}\left(t\right)$ and $C_{n\alpha }\left(t\right)$, we also calculated the temporal cross-correlation functions, e.g., $\langle C_{n\alpha }\left(t\right)C_{m\beta }\left(t\right)\rangle$ for α≠β and/or m≠n. There are 3(N−1) Rouse internal modes and therefore $9{\left(N-1\right)}^{2}$ Rouse-Rouse self- and cross-correlation functions. In Rouse’s original model, if either α≠β or m≠n or both, the temporal cross-correlation function vanishes.

How does one show that an object is performing whole-body rotation? For a fluid velocity in the *x* direction, with a non-zero velocity shear gradient $dv_{x}/dy$, the induced angular velocity should, on average, be parallel to the *z*-axis. A simple test is advanced. If the beads are each taken to be performing circular motion, the instantaneous angular rotation can be written

(12) $$\sum_{i=1}^{N}R_{i}\times v_{i}=\sum_{i=1}^{N}R_{i}\times \left(\Omega \times R_{i}\right).$$

The *z* component of L is $\hat{k}\cdot L$. Applying standard identities, one obtains for the rotational velocity $\Omega =\omega_{z}\hat{k}$ around the *z*-axis

(13) $$\omega_{z}\sum_{i=1}^{N}\left(\langle {\left(x_{i}\right)}^{2}\rangle +\langle {\left(y_{i}\right)}^{2}\rangle \right)=\langle \sum_{i=1}^{N}x_{i}\frac{dy_{i}}{dt}-\sum_{i=1}^{N}y_{i}\frac{dx_{i}}{dt}\rangle .$$

Corresponding forms describe rotation around the *x* and *y* axes. The velocities are related to the bead displacements during a single time step Δt, namely bead *i*’s displacements are $\Delta x_{i}=\Delta t\;dx_{i}/dt$, $\Delta y_{i}=\Delta t\;dy_{i}/dt$, and $\Delta z_{i}=\Delta t\;dz_{i}/dt$, so in evaluating the right-hand-side of Equation (13) we replace the velocities with the single-step displacements.

For whole-body rotation, the two terms on the right-hand-side of Equation (13) are equal by symmetry. In the absence of rotation, the first sum on the right hand side of the equation will average to zero. A polymer chain is not a solid object that performs rigid-body motion, so Ω should not be overinterpreted. Sablic, et al. [6] discuss rotation in terms of Eckart frames, and note alternative definitions of rotation rates and their physical implications.

Simulations were made for 8 and 16 bead chains at shear rates G∈(0,0.15); we treat here outcomes from 16 bead chains. In the simulations, we chose k=1, f=1, nominal temperature $k_{B}T=1$, basic time step Δt=0.001, unit diffusion step $\Delta r={\left(2k_{B}T\Delta T/f\right)}^{1/2}$, with a unit force $kr_{i,i+1}$ giving a displacement Δt/f. The characteristic functions were computed every ten time steps. A simulation with Δt=0.0003 gave very nearly the same results as a simulation using the longer time step.

Calculations were performed on an 8-core 3.4 Ghz CPU and an Nvidia Tesla K-40 GPU using locally written software run under Simply Fortran 2 and PGI Fortran. In production runs, polymer positions were advanced through $1\times 10^{8}$ time steps. Prior to each production run, a $1\times 10^{7}$ or longer timestep thermalization run was performed.

## 3. Results

**Figure 1:**
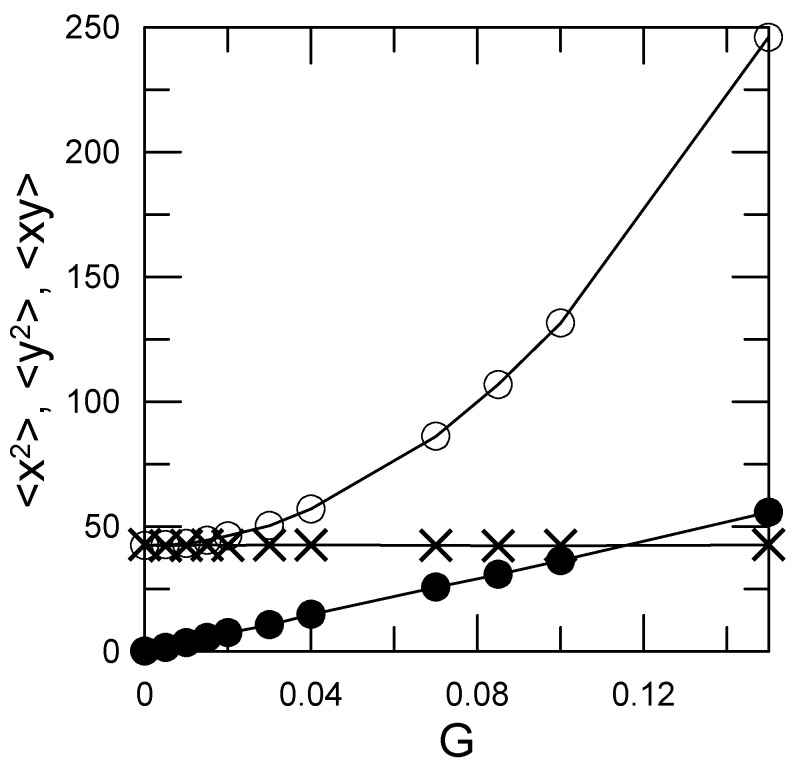
Effect of the shear rate *G* on the polymer coil shape, from the equal-time correlation functions $\langle {\left(x_{i}\right)}^{2}\rangle$ (open circles, solid line to guide the eye), $\langle {\left(y_{i}\right)}^{2}\rangle$ (crosses, line is linear fit), and $\langle x_{i}y_{i}\rangle$ (filled circles, line is linear fit).

We first consider the effect of shear on the polymer coil’s shape. As shown by Figure 1, our results include both a small-shear region, in which the polymer coil is not distorted significantly, and a large-shear region, in which the polymer coil on the average is distorted by the shear. Figure 1 plots the second moments $\langle x_{i}^{2}\rangle$, $\langle y_{i}^{2}\rangle$, and $\langle x_{i}y_{i}\rangle$ against shear rate. At zero shear, $\langle x_{i}^{2}\rangle =\langle y_{i}^{2}\rangle$. With increasing shear, the polymer is stretched in the *x* direction, but not in the *y* or (not shown) *z* directions, so that $\langle x_{i}^{2}\rangle >\langle y_{i}^{2}\rangle$. The shear field creates a non-zero $\langle x_{i}y_{i}\rangle$ correlation that increases nearly linearly with shear rate *G*. However, a shear $dv_{x}/dy$ has no effect on bead displacement in the *z* direction, so $\langle y_{i}z_{i}\rangle$ and $\langle x_{i}z_{i}\rangle$ remain equal to zero regardless of the shear rate.

We now examine the most fundamental question. Do simulated chains rotate when placed in a shear field? Equation (13) supplies the test. When the shear rate is greater than zero, the right-hand-side of Equation (13) is non-zero. The polymer chair therefore rotates around the *z*-axis. We also evaluated the analogs of Equation (13) for rotation around the *x* and *y* axes. Our shear field creates no rotation around the *x* or *y* axes, to within the accuracy of the simulation. For G>0, rotation in the x−y plane should be clockwise, i.e., $\omega_{z}<0$, as is found.

**Figure 2:**
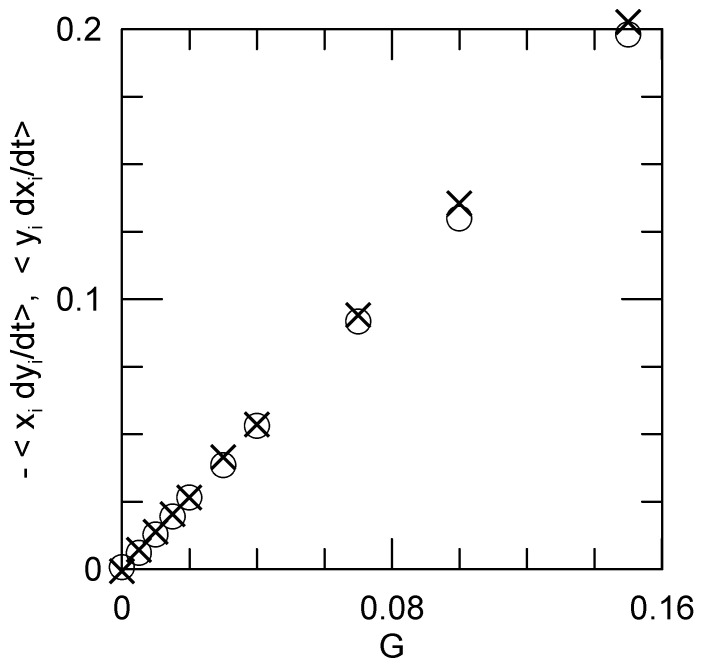
The $\sum_{i=1}^{N}x_{i}\;dy_{i}/dt$ (∘) and $\sum_{i=1}^{N}y_{i}\;dx_{i}/dt$ (×) contributions to *L* as determined at various shear rates *G*. As shown in Equation (14), for rotational motion the two terms should be equal, as seen here to good approximation.

Is the motion actually rotational? For circular motion, the two terms on the right-hand-side of Equation (13) should average to the same value. Figure 2 shows that they do. We find

(14) $$\langle \sum_{i=1}^{N}x_{i}\frac{dy_{i}}{dt}\rangle =-\langle \sum_{i=1}^{N}y_{i}\frac{dx_{i}}{dt}\rangle .$$

Rouse predicts that the left hand side of Equation (14) vanishes; it does not vanish.

The nominal angular motion *L* is

(15) $$L=\langle \sum_{i=1}^{N}x_{i}\frac{dy_{i}}{dt}\rangle -\langle \sum_{i=1}^{N}y_{i}\frac{dx_{i}}{dt}\rangle .$$

**Figure 3:**
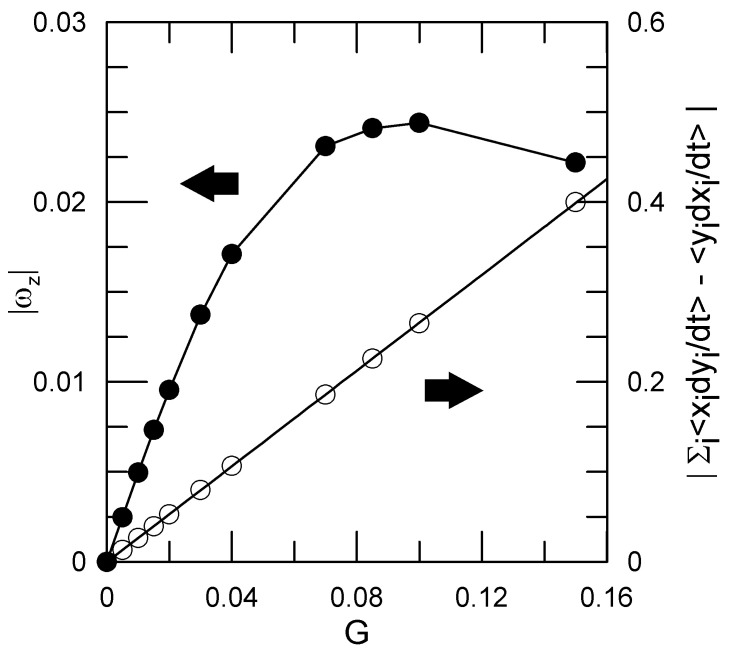
Nominal angular motion |L| (Equation (15), open circles, linear fit) and angular rotation rate $|\omega_{z}|$ (Equation (13), filled circles, line to guide the eye) for the 16-bead chain, as induced by the applied shear.

Figure 3 gives *L* and the rotation rate $\omega_{z}$ (Equation (13)), as functions of the applied shear *G*. *L* is precisely linear in *G* up to the largest *G* that we examined, as predicted by Kirkwood and Riseman. Because the polymer coil distorts when a shear is applied, *L* and $\omega_{z}$ are not simply linearly proportional to each other.

We now go beyond Kirkwood and Riseman, and beyond Rouse. Kirkwood and Riseman partitioned chain motions in shear into uniform translation, whole-body rotation, and residual contributions of internal modes. They ignored internal modes. In the Rouse model, mode relaxations are not perturbed by an applied shear. Here, we ask whether an applied shear actually affects the internal modes, as represented by the Rouse amplitudes $C_{n\alpha }\left(t\right)$ and their relaxation rates $\Gamma_{n\alpha }$. Rouse’s solutions indicate

(16) $$\langle C_{n\alpha }\left(0\right)C_{m\beta }\left(t\right)\rangle =\delta_{mn}\delta_{\alpha \beta }\langle {\left(C_{n\alpha }\left(0\right)\right)}^{2}\rangle \exp\left(-\Gamma_{n\alpha }t\right),$$

where the relaxation rate is allowed to have different values for α=x,y, or *z*. According to Rouse’s analysis, the fluctuating amplitudes $C_{n\alpha }\left(t\right)$ are uncorrelated. Modes with different *n* fluctuate independently of each other. Modes with the same *n*, but corresponding to different directions (different α), also fluctuate independently. The temporal correlation function for each mode decays exponentially in time; its relaxation rate $\Gamma_{n}$ is independent of the applied shear.

**Figure 4:**
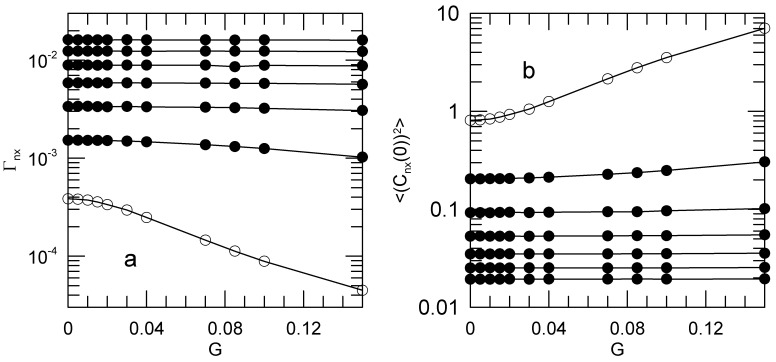
Effect of shear rate *G* on the Rouse-Rouse time correlation functions $\langle C_{nx}\left(0\right)C_{nx}\left(t\right)\rangle$, showing the dependences of (**a**) $\Gamma_{nx}$ and (**b**) $\langle {\left(C_{nx}\left(0\right)\right)}^{2}\rangle$ on *G*, the *x* in $\Gamma_{nx}$ corresponding to $\langle C_{nx}\left(0\right)C_{nx}\left(t\right)\rangle$. The open circles denote the n=1 mode, the n=2 to n=7 modes (filled circles) moving seriatim away from the n=1 mode’s behavior. $\Gamma_{1x}$ depends on *G* down to the smallest non-zero *G* that we studied. Lines serve to guide the eye.

We first consider the autocorrelation functions $\langle C_{n\alpha }\left(0\right)C_{n\alpha }\left(t\right)\rangle$. We obtained the $\Gamma_{n\alpha }$ and $\langle {\left(C_{nx}\left(0\right)\right)}^{2}\rangle$ as functions of the shear rate by fitting an early-time segment of each $\langle C_{nx}\left(0\right)C_{n\alpha }\left(t\right)\rangle$ to a single exponential. Figure 4a shows the decay rates $\Gamma_{nx}$ as functions of the shear rate. Open circles mark the n=1 mode. Figure 4b shows the corresponding mean-square average amplitudes $\langle {\left(C_{nx}\left(0\right)\right)}^{2}\rangle$.

For some modes, the decay rates and initial amplitudes are significantly shear-sensitive. For n=3 and 2, and much more markedly for n=1, the mode relaxation rates $\Gamma_{nx}$ decrease with increasing shear rate, while the corresponding mode amplitudes $\langle {\left(C_{nx}\left(0\right)\right)}^{2}\rangle$ increase with increasing shear rate. For n>3, $\Gamma_{nx}$ and $\langle {\left(C_{nx}\left(0\right)\right)}^{2}\rangle$ are very nearly independent of shear rate. The relaxation rates and amplitudes for the *y* and *z* components of the Rouse modes are independent of the shear rate. We did not explore the dependence of this result on chain length. These non-trivial dependences of the mode amplitudes and relaxation rates on shear rate are contrary to Rouse’s picture, in which the $\Gamma_{nx}$ and $\langle {\left(C_{nx}\left(0\right)\right)}^{2}\rangle$ are not affected by solvent shear.

**Figure 5:**
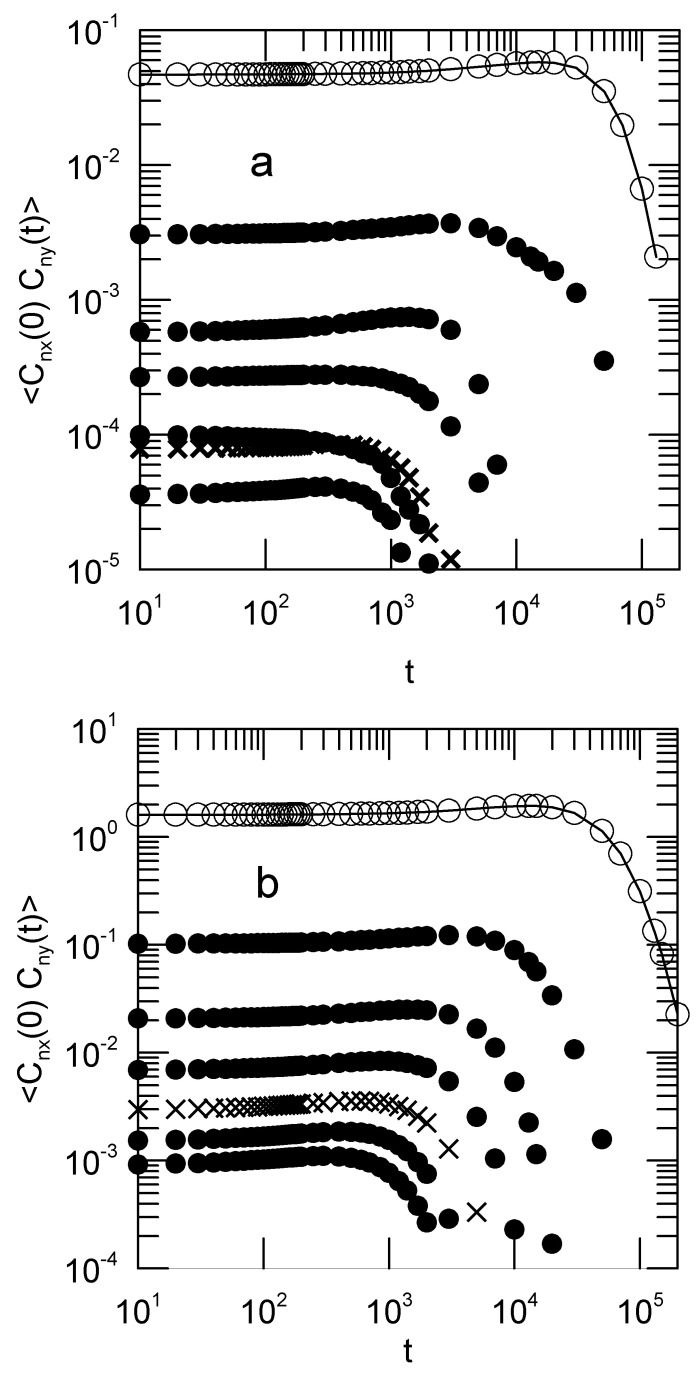
The Rouse-Rouse cross-correlation functions $\langle C_{nx}\left(0\right)C_{ny}\left(t\right)\rangle$ for a polymer coil in shear with (**a**) G=0.005 and (**b**) G=0.150. At non-zero shear rates, the Rouse modes become cross-correlated. Open circles mark n=1, with lines to guide the eye, with n=2 to 7 moving seriatim away from the n=1 line. Crosses are (**a**) n=6 and (**b**) n=5;. Note the large change in the vertical scale between these two figures.

When shear is applied, some Rouse modes become cross-correlated. Figure 5 shows the xy cross-correlations $\langle C_{nx}\left(0\right)C_{ny}\left(t\right)\rangle$. These cross-correlation functions vanish in the Rouse model. They are not zero in our simulations. The corresponding yz and zx cross-correlation functions (not shown) do vanish no matter whether or not shear is applied, as do all cross-correlation functions $\langle C_{n\alpha }\left(0\right)C_{m\beta }\left(t\right)\rangle$ with n≠m. The cross-correlation functions are not exponentials; they first increase and then fall off rapidly.

**Figure 6:**
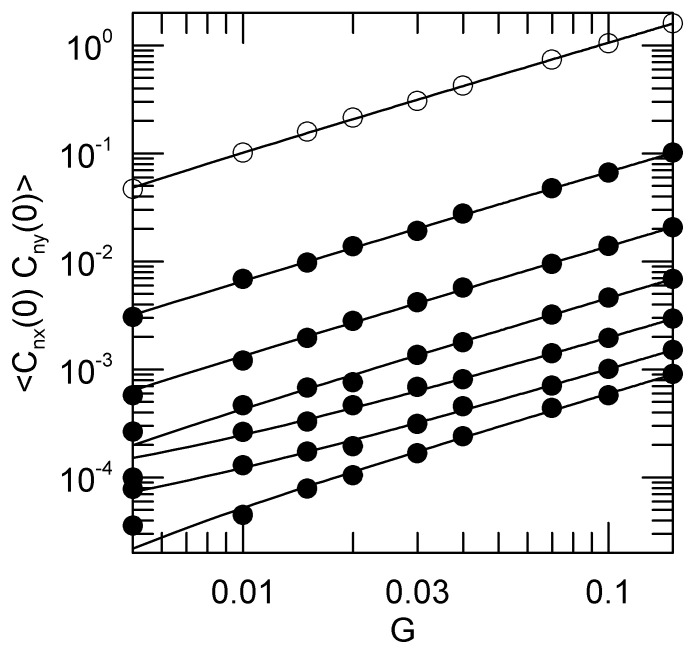
The initial amplitudes $\langle C_{nx}\left(0\right)C_{ny}\left(0\right)\rangle$ of the Rouse-Rouse cross correlation functions as functions of the shear *G*. Open circles mark the n=1 modes, with n=2 to 7 (filled circles) appearing seriatim below them. $\langle C_{nx}\left(0\right)C_{ny}\left(0\right)\rangle$ decreases monotonically with increasing *n*. Solid lines are linear fits to $\langle C_{nx}\left(0\right)C_{ny}\left(0\right)\rangle =aG+b$, *a* and *b* being fitting constants, linear fits not being straight lines on a log-log plot.

The time dependences of the $\langle C_{nx}\left(0\right)C_{ny}\left(t\right)\rangle$ are qualitatively only little affected by the shear rate, but the initial amplitudes $\langle C_{nx}\left(0\right)C_{ny}\left(0\right)\rangle$ depend strongly on *G*. Figure 6 shows this dependence. To reasonable approximation, the initial amplitudes of the cross-correlation functions are linear in the shear rate *G*.

The x–y correlations are clearly driven by rotation. Rotational motion around the *z*-axis will pump amplitude directly from $C_{nx}$ into the corresponding $C_{ny}$, as may be seen by considering the rotation of a perfectly rigid body. Whatever the amplitude $C_{nx}$ was at time 0, at the moment the rigid body has rotated through 90 degrees the component $C_{ny}$ is exactly equal in magnitude to the initial component $C_{nx}$. If the $C_{nx}$ and $C_{ny}$ were initially uncorrelated, rotation will cause the cross-correlation functions $\langle C_{nx}\left(0\right)C_{ny}\left(t\right)\rangle$ to increase with increasing time. Indeed, a close examination of the cross-correlation functions in Figure 5 suggests the presence of such an increase at longer times.

Whether or not they are normal modes of the system, the Rouse modes, interpreted as collective coordinates, provide a complete description of the bead positions at any time. However, if the Rouse coordinates are not normal modes, it becomes interesting to examine alternative sets of collective coordinates that describe the polymer’s conformation. Here, we examine Fourier components and wavelet components.

**Figure 7:**
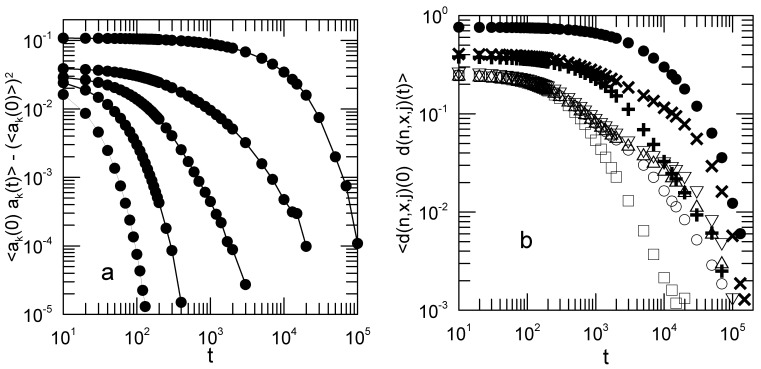
Temporal autocorrelation functions for (**a**) several spatial Fourier components of the bead positions, with lines to guide the eye, and (**b**) a Haar-like wavelet decomposition of the particle positions. These representative results refer to a 16-bead chain and G=0.03. The wavelet components are d(1,x,1)(□), d(1,x,2)(∘), d(1,x,3)(Δ), d(1,x,4)(▿), d(2,x,1)(+), d(2,x,2)(×), and d(3,x,1)(•).

Figure 7 shows representative measurements of two sets of collective coordinates that could be used as alternatives to Rouse coordinates. It is not claimed that either of these sets is necessarily the best possible choice for a set of collective coordinates, but only that there are alternatives to Rouse’s coordinates that may be worth examining. The polymer coil had 16 beads; the shear rate was 0.03. The spatial Fourier components do not decay as simple exponentials, in that they decay too slowly at longer times, but there is no sign in them of multimodal behavior.

Wavelet decompositions provide measurements of true localized motions, showing that even in this extremely simple model for polymer dynamics there is room for local differentiation of structural relaxation. We show here only the components corresponding to one-half of the full chain; the corresponding components for the other half of the chain show exactly the same set of behaviors. In considering the series d(1,x,j) (open symbols) for j∈(1,4), d(1,x,1), which relaxes the most rapidly, corresponds to the motions of the outer pair of beads. The d(1,x,j) for j>2 have clearly bimodal relaxations, speaking to more complex chain dynamics nearer to the center of the polymer. The d(2,x,j) emphasize the differences between inner and outer beads of the polymer. d(2,x,1), which corresponds to the outer four beads of the polymer, has a non-exponential but unimodal relaxation; d(2,x,2), describing the four beads nearest the chain center, shows a visibly bimodal relaxation. For d(1,x,j) and d(2,x,j), the j=1 function decays more rapidly, showing directly that the outer beads are more mobile.

## 4. Discussion

This paper describes a simulational study of the motions of a polymer in a shear field. Comparison was made with the Kirkwood–Riseman and Rouse treatments of the dynamics of an isolated polymer chain. We show that the Kirkwood–Riseman model of polymer dynamics, in which a polymer coil translates and rotates when subject to the influence of a shear, is qualitatively correct. The Rouse model, in which polymer coils do not rotate during viscometric studies, is incorrect as applied to the viscosity increment of a polymer in solution. We note several alternatives to Rouse coordinates that could, in principle, serve as descriptions of polymer internal motions.

We return to the observation that the Rouse and Kirkwood–Riseman models do not agree as to how many internal modes a polymer molecule has, namely 3N−3 or 3N−6, respectively. A Rouse chain is not in a shear field, so it does not perform driven rotation, but it is not held in place; it is not constrained not to rotate. Orthodox classical mechanics, not to mention the theory of molecular vibrations as seen in infrared and Raman spectroscopy, immediately tell us that any N-atom molecule that is free to move has three translational modes, three rotational modes, and 3N−6 internal modes. The Rouse model finds 3N−3 internal modes, so it must be inconsistent with classical mechanics.

Is the lack of rotation in the Rouse model important? The Rouse model provides us with formulae for the end-to-end relaxation time and the viscosity increment due to a polymer molecule, each phrased entirely in terms of the relaxation times of the polymer’s 3N−3 internal modes. However, those formulae refer to particular mechanical models of the polymer. In the Rouse model, the shear field assumed above creates an affine displacement of the polymer beads parallel to the *x*-axis. The beads are then stationary, the hydrodynamic force on the beads due to the fluid motion being exactly cancelled by the restoring spring forces due to the Hookean springs connecting the beads. If the polymer is rotating, its beads are moving with respect to the solvent in ways not described an affine displacement, meaning that the dissipation due to the polymer includes terms not obtained from the Rouse mechanical picture. The Rouse viscosity formula is then incorrect. Furthermore, the lack of rotation leads to a peculiar picture of relaxation of the end-to-end vector. Suppose at some moment exactly one Rouse mode is non-zero, so that the beads are spread out along a single axis, *x* or *y* or *z*. The polymer relaxes by pulling all beads in toward the center of mass. Contrary to reasonable expectations, in this case rotational diffusion plays no role in relaxing the end-to-end vector.

It is certainly legitimate to ask how the issues raised here were not already noticed. It is not suggested here that there were past errors. Several contributory factors are readily identified. First, while there are multiple excellent presentations of the Rouse–Zimm model, e.g., refs. [5,6], equivalent presentations of the Kirkwood–Riseman model more recent than their original paper are far less common, so there is little familiarity with the Kirkwood–Riseman model. Second, in the absence of shear, the two models converge; computer simulations of polymer coils in unsheared liquids cannot readily reveal the disagreement between the models as to their creation of shear viscosity. Third, in order to identify our issues, one would have needed to analyse a chain trajectory with the correct diagnostic, e.g., Equation (14), but in the context of the Rouse–Zimm model there is no rational reason to develop such a diagnostic. As a result, in past studies many fine questions have been asked about the nature of polymer dynamics, but not the questions answered here.

Larson and co-workers [7,8] provide considerable evidence that potential energies more precise than Rouse’s potential can cause a chain’s dynamics to deviate from simple Rouse behavior. Jain and Larson [7] made Brownian dynamics simulations of a string of polymer beads to which stiff springs, bond-angle, and bond-torsion-angle forces were added seriatim. They calculated the time autocorrelation functions for the polymer end-to-end vector and the connector unit-vector autocorrelation functions, the latter being averaged over all springs in the chain. Dalal and Larson [8] extended these results, showing that adding side groups, chain excluded-volume effects, and explicit treatment of solvent molecules jointly lead to the experimentally-observed single-exponential relaxation for short chains. They also note what they viewed as an interesting coincidence, namely that the relaxation times for the orientation of the chain end-to-end vector and the single-spring orientation vectors are very nearly the same.

The difficulty with the Rouse model is apparent in Equations (1)–(3). These equations include a solvent shear force $Gy_{i}\hat{i}$ on each bead. That force is absent from equations solved by Rouse. However, Rouse calculated how a bead-spring polymer coil would evolve in time in a quiescent fluid. In a quiescent fluid, the polymer coil by symmetry has no tendency to rotate. When a fluid shear field $Gy_{i}\hat{i}$ is included in the calculation, the forces on the beads are changed. The motions of the beads therefore also change, and are no longer the motions described by Rouse. The Rouse model thus does not describe polymer dynamics during a rheological experiment. In his original paper, Rouse uses his quiescent-fluid solutions to calculate dissipation and hence the viscosity increment for a polymer in a shear flow, even though his solutions are not applicable when the polymer is in a shear field and thus contributing to viscous dissipation.

The above has focused on a polymer coil in an imposed macroscopic shear field, as encountered in viscoelastic measurements. However, the fluctuation–dissipation theorem gives us two other circumstances in which polymer coils find themselves in shear fields:

First, consider a polymer coil performing Brownian motion. The fluctuation–dissipation theorem indicates that if the chain diffuses through some distance in a given time, the chain motions will have correlated fluid motions, exactly as if the chain’s motion were being created by an imposed external force. That fluid motion, the wake created in the solvent by the polymer, acts on other polymer coils, causing them to move in turn. Because the fluid flow is not the same everywhere, those other polymer coils are subject to a fluid shear field which causes them to translate, rotate, and create fresh flow fields in the surrounding solvent. This image of flow fields being scattered and re-scattered by diffusing macromolecules forms the core of modern theoretical treatments of the diffusion of interacting spherical colloidal particles [9], these theoretical treatments giving reasonably accurate quantitative predictions for colloidal behavior. It should therefore not be surprising that the same general approach is valid for interacting polymer coils in solution. That is, via the fluctuation–dissipation theorem we can extend the Kirkwood–Riseman model from treating a single isolated polymer molecule to treat the hydrodynamic interactions between polymer molecules. Indeed, there is a substantial development of polymer dynamics in non-dilute solutions based on computing the hydrodynamic interactions between polymer coils [10,11].

Second, consider a polymer coil in a quiescent fluid. On average, there is no tendency for the molecule to rotate in any direction. However, the fluctuating thermal forces on the polymer beads create evanescent fluctuating torques on the molecule as a whole, causing the polymer end-to-end vector to perform rotational diffusion, so that its later positions gradually become decorrelated from its earlier positions. The end-to-end vector is a sum of the individual bead-to-bead vectors, so there is a component of each bead-to-bead vector that is correlated with the chain end-to-end vector. The bead-to-bead vectors can only become completely uncorrelated on the time scale on which the chain end-to-end vector relaxes. The result of whole-chain rotational diffusion is that the spring unit-vector correlation functions will in part relax on the time scale on which the chain end-to-end vector relaxes, precisely as found by Dalal and Larson [8].

Rouse modes and the Rouse model are used in an extremely large number of different contexts. I have not here generated a full list of contexts in which the Rouse model is inappropriate, though clearly any theoretical problem in which a polymer chain is placed in a shear field must be on that list; nor have I considered here any extensions to the Kirkwood–Riseman model.

## Data Availability

All data is in the main body of the paper.

## Supplementary Materials

The following supporting information can be downloaded at: https://www.mdpi.com/article/10.3390/polym15091995/s1, File S1. The Kirkwood-Riseman Model of Polymer Solution Dynamics is Qualitatively Correct Supplemental Information The software used for these calculations in available to the public. I posted in November 2017 five files that jointly created these simulations. (i) RouseRot009 is the actual uncompiled computer code. A standard Fortran compiler should handle it (www.researchgate.net/publication/320991192_rouserot009); (ii) Rousevariables is the Include file setting up the variables, e.g., matrix dimensions. You need it to compile RouseRot009 (www.researchgate.net/publication/320991239_rousevariables03); (iii) ControlP2 sets specifications for a single simulation run, e.g., how many beads there are: (www.researchgate.net/publication/320991107_controlp2); (iv) Retain can hold a set of thermalized bead positions that can be used as input next time (www.researchgate.net/publication/320991236_retain); (v) Random are the seeds for the random number generator, taken as the last group of seeds in use during the last run (www.researchgate.net/publication/320991106_random).

## References

[1] Kirkwood J.G., Riseman J. (1948). The Intrinsic Viscosities and Diffusion Coefficients of Flexible Molecules in Solution. J. Chem. Phys..

[2] Rouse P.E. (1953). A Theory of the Linear Viscoelastic Properties of Dilute Solutions of Coiling Polymers. J. Chem. Phys..

[3] Zimm B.H. (1956). Dynamics of Polymer Molecules in Dilute Solution: Viscoelasticity, Flow Birefringence, and Dielectric Loss. J. Chem. Phys..

[4] Haar A. (1910). Zur Theorie der orthogonalen Funktionensysteme. Math. Ann..

[5] Daubechies I. (1992). Ten Lectures on Wavelets. CBMS-NSF Regional Conference Series in Applied Math.

[6] Sablic J., Delgado-Buscalioi R., Praprotnick M. (2017). Application of the Eckart Frame to Soft Matter: Rotation of Star Polymers under Shear Flow. Cornell University Library. arXiv.

[7] Jain S., Larson R.G. (2008). Effects of Bending and Torsional Potentials on High-Frequency Viscoelasticity of Dilute Polymer Solutions. Macromolecules.

[8] Dalal I.S., Larson R.G. (2013). Explaining the Absence of High-Frequency Viscoelastic Relaxation modes of Polymers in Dilute Solution. Macromolecules.

[9] Phillies G.D.J. (2016). Diffusion in Crowded Solutions. Adv. Chem. Phys..

[10] Phillies G.D.J. (2016). The Hydrodynamic Scaling Model for the Dynamics of Non-Dilute Polymer Solutions: A Comprehensive Review. Cornell University Library. http://arxiv.orgarxiv.org/abs/1606.09302.

[11] Phillies G.D.J. (2002). Self-Consistency of Hydrodynamic Models for the Low-Shear Viscosity and the Self-Diffusion Coefficient. Macromolecules.

