# Supplementary material for: The Kirkwood–Riseman Model of Polymer Solution Dynamics Is Qualitatively Correct
Source: Polymers (Basel). 2023 Apr 23;15(9):1995. doi: 10.3390/polym15091995 (PMC10180932; doi:10.3390/polym15091995)
Supplement: Supplementary file 1 [file polymers-15-01995-s001.zip › polymers-2299888-supplementary.pdf]

polymers

MDPI

Article

The Kirkwood-Riseman Model of Polymer Solution Dynamics is Qualitatively Correct Supplemental Information

George David Joseph Phillies

Department of Physics, Worcester Polytechnic Institute, Worcester, MA 01609-2280, USA; phillies@4liberty.net; Tel.: +1-508-754-1859

Abstract: The Supplemental Information provides a detailed description of the Rouse and Kirkwood-Riseman models.

check for updates

Citation: Phillies, G.D.J. The Kirkwood-Riseman Model of Polymer Solution Dynamics is Qualitatively Correct Supplemental Information. *Polymers* **2023**, *15*, 1995. https://doi.org/polym15091995

Academic Editor: Pavlos Stephanou

Received: 8 March 2023
Revised: 15 April 2023
Accepted: 19 April 2023
Published: 23 April 2023

Copyright: © 2023 by the author. Licensee MDPI, Basel, Switzerland. This article is an open access article distributed under the terms and conditions of the Creative Commons Attribution (CC BY) license (https://creativecommons.org/licenses/by/4.0/).

The Supplemental Materials present aspects of the Rouse[1] and Kirkwood-Riseman[2] models. We begin with the more familiar Rouse model, and then consider the Kirkwood-Riseman model.

Rouse's original treatment was quite involved. As is often the case with novel theoretical results, as time advances the key aspects of the calculation are abstracted from the original structure. The presentation of Doi and Edwards[3] and the more extended development by Padding[4] are followed here. The Rouse model describes an isolated polymer in a solvent. The polymer is approximated as a linear sequence of N beads, each linked to the next by a springlike connector. The bead positions are denoted $(\mathbf{R}_1, \mathbf{R}_2, \ldots \mathbf{R}_N)$. The beads are points having no excluded volume; they are all free to move with respect to each other. Each bead has a hydrodynamic drag coefficient f. The connectors do not interact with the solvent.

The strength of the connectors is determined by the Gaussian statistics that describe the shape of a random-walk polymer coil. In Rouse's model, each bead represents some substantial number of monomers. The distance along the polymer chain from each bead to the next is sufficiently large that the bead-bead distances $r_{i,i+1} = |\mathbf{R}_{i+1} - \mathbf{R}_i|$ have Gaussian distributions $P(r_{i,i+1}) \sim \exp(-\alpha r_{i,i+1}^2)$.

Rouse implicitly explains that for each statistico-mechanical distribution function $P(r_{ij})$, there is a corresponding potential of average force $W(r_{ij})$, namely

$$W(r_{ij}) = -k_B T \ln(P(r_{ij})). \tag{S1}$$

Here k_B is Boltzmann's constant and T is the absolute temperature. The potential of average force gives the average force between two adjoining beads that are a distance r_{ij} apart.

The calculations here use Rouse's original potential of average force

$$W(r_{ij}) = \frac{1}{2} k r_{ij}^2, \tag{S2}$$

for two beads i and j that adjoin along the polymer chain. In Rouse's model, the force constant k is determined by the mean-square bead separation b, namely

$$k = \frac{3k_B T}{b^2}. \tag{S3}$$

It is possible to use considerably more sophisticated forms for the potential energy of the polymer chain. Note, for example, work of Tsalikis, et al.[5], Perez-Aparicio, et al.[6], and Kalathi, et al.[7]. However, the objective here is to test the Rouse and Kirkwood-Riseman models. To test the Rouse model, we must use Rouse's potential. The Kirkwood-Riseman model specifies only average interbead distances, and does not invoke a particular

form for the interbead potential energy. In the original Rouse model, a bead i was also subject to a thermal force $\mathcal{F}_i(t)$ due to fluctuations in the solvent. Hydrodynamic interactions between beads, and correlations between the thermal forces on different beads, are neglected in the Rouse model but included in the elsewise-similar Zimm[8] model.

We can now write the equations of motion – Newton's second law – for each bead of the Rouse model. The drag force on each bead is large. On the time scales of interest bead motions are massively overdamped. Bead inertia is therefore neglected. If bead inertia vanishes, the total force on each bead must also vanish. The direct forces (the spring forces) on each bead must therefore cancel the hydrodynamic forces. The equations of motion for beads other than the two end beads (beads 1 and N) are then

$$f\frac{d\mathbf{R}_i}{dt} = -k(2\mathbf{R}_i - \mathbf{R}_{i-1} - \mathbf{R}_{i+1}) + \mathcal{F}_i(t), \tag{S4}$$

while for the first and last beads in the chain one has

$$f\frac{d\mathbf{R}_1}{dt} = -k(\mathbf{R}_1 - \mathbf{R}_2) + \mathcal{F}_1(t), \tag{S5}$$

and

$$f\frac{d\mathbf{R}_N}{dt} = -k(\mathbf{R}_N - \mathbf{R}_{N-1}) + \mathcal{F}_N(t). \tag{S6}$$

It is generally the case that the bead positions $\mathbf{R}_i$ are not all equal to each other. As a result, the spring forces on individual beads are not zero, so in general the beads must be moving with respect to the solvent to create the countervailing hydrodynamic forces. However, if the thermal forces driving fluctuations are silenced, one obtains the equilibrium rest conformation of the Rouse model, namely a mathematical point with all beads in the same location.

The above are N vector equations. They correspond to a total of $3N$ scalar equations describing bead motions. The direction cosine for the x-component of the force between beads i and $i+1$ is $(x_{i+1} - x_i)/|\mathbf{R}_{i+1} - \mathbf{R}_i|$. Corresponding forms give the y and z direction cosines for each force vector. The N vector equations given above therefore correspond to $3N$ scalar equations such as

$$f\frac{dx_i}{dt} = -k(2x_i - x_{i-1} - x_{i+1}) + \mathcal{F}_{xi}, \tag{S7}$$

x_i being the x-component of the bead coordinate of bead i and $\mathcal{F}_{xi}$ being the x component of the thermal force on bead i. As explained by Rouse in his original paper, the equations for the x coordinates, for the y coordinates, and for the z coordinates are, except for the coordinate label, the same as each other. Changing the x-component of a particle's position has no effect on the y and z components of the forces on any particle, and correspondingly for displacements of a bead in the y or z directions. The equations of motion for the x, y, and z coordinates are thus completely uncoupled. The equations of motion therefore partition into three sets of N coupled equations, one set for each of the three coordinate axes. Because each set of equations is the same as the others, except for the label on the coordinates, only one set of N equations needs to be solved. The solutions for the other two sets of equations can be obtained by a change of the coordinate label. While the equations of motion of the beads do partition into three sets of N equations, one set for each dimension, the model is three-dimensional, not one-dimensional. Beads move in all three coordinate directions.

Equation S7 and the matching equations for beads 1 and N are a set of N coupled linear differential equations whose coefficients are constants. The solutions are therefore a set of N eigenmodes Q_n describing motions parallel to one of the three coordinate axes, each mode having a corresponding eigenvalue Γ_n. One mode has eigenvalue $\Gamma_0 = 0$; that mode corresponds to the center-of-mass location of the polymer, its time derivative representing uniform translation of all beads with the same speed in the same direction.

The other $N-1$ modes decay exponentially ($\exp(-\Gamma_n t)$) in time; their relaxation rates Γ_n are

$$\Gamma_n = \frac{8k\sin^2(n\pi/2N)}{f} \tag{S8}$$

with $n \in (1, N-1)$ being the mode label.

The normal mode amplitudes $C_{nx}(t)$ for the x-coordinate modes may be calculated from the bead coordinates $x_i(t)$ via

$$C_{nx}(t) = \frac{1}{N}\sum_{i=1}^{N} x_i(t)\cos\left(\frac{n\pi(i-1/2)}{N}\right). \tag{S9}$$

Entirely similar equations give the amplitudes C_{ny} and C_{nz} of the y- and z-coordinate modes. The inverse equations give the x_i in terms of the normal mode amplitudes as

$$x_i(t) = C_{0x}(t) + 2\sum_{n=1}^{N-1} C_{nx}(t)\cos\left(\frac{n\pi(i-1/2)}{N}\right). \tag{S10}$$

Totally similar equations describe the y and z modes. Standard mathematical techniques show how the random forces $\mathcal{F}_{xi}(t)$ serve as source terms, driving the fluctuations in the $C_{nx}(t)$.

There are three coordinate axes, so the relaxation rates Γ_n are three-fold degenerate. For each n, the same relaxation rate applies to all three coordinate axes. The modes having degenerate eigenvalues are orthogonal; their amplitudes fluctuate independently. The Rouse model thus has three translational modes, each with eigenvalue zero, and $3N-3$ internal modes ('internal' in the sense that in each internal mode the beads move with respect to each other as time goes on) having non-zero eigenvalues.

On setting all but one of the C_{nx} to zero, eq. S10 gives the representation in position space of the eigenvector corresponding to C_{nx}. The Rouse eigenvectors thus provide a set of $3N$ normal coordinates that can replace the bead coordinates $\{x_i, y_i, z_i\}$ as a specification of the polymer's configuration. Eqs. S9 and S10 may also be interpreted as a pair of discrete Fourier transforms, in which $i-1/2$ plays the role of the position coordinate, $n\pi/N$ is the wave vector, and x_i and C_{nx} are the amplitudes of the function and its transform at i and n, respectively.

Rouse uses the Rouse modes to describe the behavior of a polymer coil in a shear flow. In Rouse's calculation, a shear flow exerts forces on the polymer. The polymer's responses are described by the Rouse modes. The Rouse solutions therefore were taken by Rouse to be valid descriptions of polymer motion when a shear flow is applied.

Polymer coils whose motions are described by Rouse's model have one ill-recognized property: They do not rotate. This property follows by comparison with a standard problem in classical mechanics, namely the vibrational modes of an isolated molecule. In general, an N-atom molecule has 3 translational modes with eigenvalue zero, 3 rotational modes with eigenvalue zero, and $3N-6$ internal vibrational modes. The internal modes are the modes that change the distances between pairs of atoms. In translation and rotation the distances between the atoms remain fixed. The Rouse problem only differs from the molecular vibration problem in that the Rouse equations of motion are overdamped, so the Rouse amplitudes relax exponentially at some rate Γ_n rather than oscillating at some frequency ω_n. A polymer coil is therefore like a vibrating isolated molecule in having a total of $3N$ modes. However, the $3N$ modes of the Rouse model include 3 translational modes and $3N-3$ internal modes, for a total of $3N$ modes, leaving no modes available for rotational motion. We return to this issue in the discussion.

The statement that Rouse chains do not rotate is not new. Rouse[1] specifies in his paper that a polymer coil under shear does not rotate, namely (his paper, p. 1274, column 2) "...since the velocity of the liquid has a nonvanishing component only in the x direction, the components $(\dot{y}_j)_\alpha$ and $(\dot{z}_j)_\alpha$ are zero." $(\dot{y}_j)_\alpha$ and $(\dot{z}_j)_\alpha$ are the velocities of bead j in the y and

z directions due to the shear. If the chain is rotating, either $(\dot{y}_j)_\alpha$ or $(\dot{z}_j)_\alpha$ must be non-zero. Rouse also argues his paper (p. 1274, column 2, top) that '...an atom at the junction between two submolecules...' (springs) moves '...with a velocity equal to that of the surrounding liquid...' except for Brownian motion, because, according to Rouse, otherwise there would be motion of the solvent relative to the polymer chain, leading to energy dissipation. If the beads only move with the liquid, then they can only be moving parallel to the x-axis.

We now consider the Kirkwood-Riseman model[2]. While both models refer to an unbranched linear chain of beads, the Kirkwood-Riseman model is radically different from the Rouse model. The Kirkwood-Riseman model is based on three fundamental assumptions. First, all distances between pairs of beads are treated as being their statistico-mechanical average values; fluctuations and changes in these distances are explicitly not included in the model. Second, the distribution of beads around the chain center-of-mess is spherically symmetric. Third, the system is massively overdamped, so that the inertia of the polymer coil is negligible. These three assumptions completely define the chain dynamics, the description of how a Kirkwood-Riseman polymer chain moves in solution. Kirkwood and Riseman recognized that a polymer coil has internal modes ("fluctuations") so that polymer beads actually do move with respect to each other, but these bead motions were specified as being not included in their model.

The system is heavily overdamped, so its inertia is negligible. The total force on the chain must therefore be zero. The moments of inertia of the chain are negligible. The total torque on the chain must therefore also be zero. A chain satisfies these two zero conditions by adjusting its linear velocity $\mathbf{V}$ and its angular velocity Ω until the total force and the total torque on the chain both vanish.

Kirkwood and Riseman consider how a polymer coil moves in a shear field in which the fluid velocity is

$$\mathbf{u}_i = \mathbf{u}_i^{(0)} + G y_i \hat{\mathbf{i}}, \tag{S11}$$

Here $\mathbf{u}_i^{(0)}$ is a possible uniform motion of the fluid, G is a constant linear shear gradient, y_i is the y-component of the vector location of bead i, and $\hat{\mathbf{i}}$ is the unit vector parallel to the x-axis. We do not follow Kirkwood's notation closely. Kirkwood and Riseman included in their model bead-bead hydrodynamic interactions as described by the Oseen tensor. We return to these internal interactions below.

The Kirkwood-Riseman model describes a chain of N beads. The beads have coordinates $(\mathbf{R}_1, \mathbf{R}_2, \ldots \mathbf{R}_N)$. Sequential bead positions form a highly restricted random walk. Each bead is subject to a hydrodynamic force $\mathbf{F}_{iH}$ exerted by the fluid, and to forces due to the bonds connecting that bead to its neighbors along the chain. $\mathbf{F}_{iH}$ is determined by the bead drag coefficient f, the velocity $\mathbf{v}_i$ of the bead, and the velocity $\mathbf{u}_i$ that the fluid would have had, at the location of bead i, if bead i were not there, via

$$\mathbf{F}_{iH} = f(\mathbf{u}_i - \mathbf{v}_i). \tag{S12}$$

$\mathbf{F}_{iH}$ is the hydrodynamic force on the bead, not the total force. The total force on each bead, including the forces due to links to adjoining beads, vanishes, so $\mathbf{F}_{iH}$ in general is non-zero. Correspondingly, the bead and solvent velocities are in general not equal to each other.

In the Kirkwood-Riseman model, the velocity of bead i is

$$\mathbf{v}_i = \mathbf{V} + \Omega \times \mathbf{s}_i. \tag{S13}$$

Here $\mathbf{s}_i$ is the vector from the chain center-of-mass to bead i, V is a linear velocity, the same for each bead, and Ω is an angular rotation rate, the same for each bead. Internal modes neglected in the model would add to the right hand side of this equation an additional term $\dot{\xi}_i$, the contribution of the internal modes to the bead velocity; that term does not appear in the model.

Kirkwood and Riseman use the zero-total-force and zero-total-torque conditions to determine $\mathbf{V}$ and Ω in terms of $\mathbf{u}_i^{(0)}$ and G, finding

$$\mathbf{V} = \mathbf{u}_i^{(0)} + GY_0\hat{\mathbf{i}} \tag{S14}$$

and

$$\Omega = -\frac{G}{2}\hat{\mathbf{k}}. \tag{S15}$$

Y_0 is the y-coordinate of the polymer chain's center of mass, and $\hat{\mathbf{k}}$ is the unit vector in the z-direction. The model predicts viscous dissipation because the bead velocity $\mathbf{v}_i$ and the solvent velocity $\mathbf{u}_i$ cannot be equal at every point. For example, for most beads $\mathbf{v}_i$, but not $\mathbf{u}_i$, will have a non-zero y-component.

The calculations leading to eqs. S14 and S15 make no reference to hydrodynamic interactions between polymer beads. To calculate viscous dissipation, Kirkwood and Riseman then insert bead-bead hydrodynamic interactions. However, bead-bead hydrodynamic interactions have no effect on the dynamic model specified by eqs. S13-S15. The dynamic model is not affected by intrachain hydrodynamic interactions because bead-bead hydrodynamic interactions are *internal* forces, forces between different beads on the same chain. The total force and the total torque exerted on a polymer chain by bead-bead forces must both vanish, an outcome guaranteed by Newton's Third Law of Motion. Adding hydrodynamic interactions has no effect on the motions described by eqs. S13-S15, these being the equations that completely specify the Kirkwood-Riseman dynamic model.

In applying the Oseen tensor to describe bead-bead hydrodynamic interactions, Kirkwood and Riseman took the distance between each pair of beads to be the equilibrium average distance between those two beads. Fluctuations in those interbead distances, and the time dependences of those fluctuations, were approximated by Kirkwood and Riseman as not being important.

References

1. Rouse, P. E. A Theory of the Linear Viscoelastic Properties of Dilute Solutions of Coiling Polymers. *J. Chem. Phys.* **1953**, *21*, 1272–1280.
2. Kirkwood, J. G.; Riseman, J. The Intrinsic Viscosities and Diffusion Coefficients of Flexible Molecules in Solution. *J. Chem. Phys.* **1948**, *16*, 565–573.
3. Doi, M.; Edwards, S. F. *The Theory of Polymer Dynamics*, Oxford University Press, Oxford, United Kingdom (1986).
4. Padding, J. T.; Theory of Polymer Dynamics. https://www.scribd.com/document/181367522/THEORY-OF-POLYMER-DYNAMICS-Paddings (2005).
5. Tsalikis, D. G.; Koukoulas, T. ; Mavrantzas, V. G.; Pasquino, R.; Vlassapoulos, D.; Pyckhout-Hintzen, W.; Wischnewski, A.; Monkenbusch, M.; Richter, D. Microscopic Structure, Conformation, and Dynamics of Ring and Linear Poly(ethylene oxide) from Detailed Atomistic Molecular Dynamics Simulations: Dependence on Chain Length and Direct Comparison with Experimental Data. *Macromolecules* **2017**, *50*, 2565–2584.
6. Perez-Aparicio, R.; Alvarez, F.; Arbe, A.; Willner, L.; Richter, D.; Falus, P.; Colmenero, J. Chain Dynamics of Unentangled Poly(ethylene-alt-propylene) Melts by Means of Neutron Scattering and Fully Atomistic Molecular Dynamics Simulations. *Macromolecules* **2011**, *44*, 3129–3139.
7. Kalathi, J. T.; Kumar, S. K.; Rubinstein, M.; Grest, G. S. Rouse Mode Analysis of Chain Relaxation in Homopolymer Melts. *Macromolecules* **2014**, *47*, 6925–6931.
8. Zimm, B. H.; Dynamics of Polymer Molecules in Dilute Solution: Viscoelasticity, Flow Birefringence, and Dielectric Loss. *J. Chem. Phys.* **1956**, *24*, 269–278.
